# Teratogenicity and hyperprolactinemia

**DOI:** 10.4103/0019-5545.44909

**Published:** 2009

**Authors:** Chittaranjan Andrade

**Affiliations:** Department of Psychopharmacology, National Institute of Mental Health and Neurosciences, Bangalore 560 029, India

## CME QUESTIONS

A) Drugs should be prescribed with caution to women of childbearing age because of the risk of teratogenicity, should conception occur during treatment; this concern is particularly important when prescribing drugs that will be required for long periods, such as in the maintenance therapy of epilepsy, bipolar disorders, neuropathic pain, and migraine. Topiramate and valproate are sometimes prescribed as maintenance medications for certain or all of these indications. With this background, mark *True* or *False* against each of the following statements:

1. Topiramate monotherapy is safe during pregnancy.
2. The use of topiramate along with other antiepileptic drugs is associated with an unacceptably high teratogenic risk.
3. Valproate is one of the most teratogenic medications used in psychiatry.
4. The teratogenicity of valproate is substantially higher when the drug is used in combination with other antiepileptic medications.
5. The teratogenic risk of valproate is dose-dependent.
6. Valproate is teratogenic only during weeks 6-12 of gestation.
7. Exposure to valproate during pregnancy can be associated with low IQ in the offspring.

B) Hyperprolactinemia is a common problem with different antipsychotic drugs. Hyperprolactinemia may lead to many adverse consequences, and may thereby jeopardize drug compliance. With this background, mark *True* or *False* against each of the following statements:

1. The normal prolactin level is 25 ng/ml and above.
2. Hyperprolactinemia may result in increased libido.
3. Hyperprolactinemia may result in osteoporosis.
4. Hyperprolactinemia is uncommon with paliperidone.
5. Aripiprazole augmentation reverses antipsychotic-induced hyperprolactinemia.

## CME ANSWERS

### A) Teratogenicity

Answers: 1. False; 2. True; 3. True; 4. True; 5. True; 6. False; 7. True.

#### 1. Topiramate monotherapy during pregnancy

Topiramate is a well-established teratogen in animals such as mice, rats, and rabbits. One study[1] examined the effects of first-trimester topiramate exposure in 52 pregnancies with 41 liveborn infants. Topiramate was observed to reduce birth weight without decreasing gestational age at delivery; there was no increase in the risk of structural birth defects. However, another larger and methodologically superior study[2] documented substantial teratogenicity with the drug. This study used data from the UK Epilepsy and Pregnancy Register. The subjects were 203 women with epilepsy who had been exposed to topiramate as monotherapy (*n*=70) or as part of polytherapy (*n*=133) during the first trimester of pregnancy, and who had been referred before the outcome of pregnancy was known. There were 178 live births; the risk of major congenital malformations with topiramate was 9% (95% CI, 5.6%-14.1%) in the entire sample, and 4.8% with topiramate monotherapy. In this context, studies suggest that the risk of major malformations is 2.7-3.5% in epileptic women who do not use antiepileptic drugs during pregnancy, and those who use antiepileptic drugs in monotherapy.[3,4]

#### 2. Teratogenicity of topiramate combined with other antiepileptic drugs

In the UK Epilepsy and Pregnancy Register study[2] referred to above, major congenital malformations were more common in women exposed to topiramate combined with other antiepileptic agents (11.2%) than in those exposed to topiramate monotherapy (4.8%) during the first trimester of pregnancy. The highest risk of major malformations was when topiramate was used along with valproate (36%), or along with two or more other antiepileptic drugs (24%). Also, in women receiving topiramate in polytherapy, smallness for gestational age was associated with a higher dose of the drug.

#### 3. Teratogenicity of valproate in monotherapy

Data from the North American Antiepileptic Drug Pregnancy Registry,[4] the UK Epilepsy and Pregnancy Register,[3] and the Israeli Teratology Information Service database[5] suggest that the risk of major congenital malformations is 6-11% after first trimester exposure to valproate monotherapy. This contrasts with a risk of 2.7-3.5% in epileptic women who do not use antiepileptic medications during pregnancy, and those who use other antiepileptic monotherapy.[3,4] Data from the Australian Pregnancy Registry[6] also indicate that valproate is more teratogenic than other antiepileptic drugs.

#### 4. Teratogenicity of valproate combined with other antiepileptic drugs

Data from the UK Epilepsy and Pregnancy Register[3] showed that the risk of major congenital malformations was about 6% in women who received valproate monotherapy, and 9% in those who received valproate-containing polytherapy. The teratogenic risk was about 12.5% when valproate was combined with lamotrigine but only about 2.7% when other drugs were combined with lamotrigine.[7] The teratogenic risk was 36% with the topiramate-valproate combination but only 4.8% with topiramate monotherapy.[2] It should be remembered that some of the extreme values may be only estimates because they were obtained from relatively small samples. It should also be kept in mind that confounding by indication cannot altogether be ruled out: women receiving such combinations may have been more severely ill, and hence more likely to give birth to offspring with major malformations. However, at least one study suggests that confounding by indication is unlikely.[8]

#### 5. Valproate dose and teratogenic risk

Data from the Israeli Teratology Information Service database[5] suggested that teratogenicity with valproate was more likely at doses of 1000 mg/day or higher; the risk was 21.9% with valproate but just 2.5% in controls.[5] Data from the Australian Pregnancy Registry also indicated that the risk was greater in patients who used doses of 1100 mg/day and higher.[6] Data from the North American Antiepileptic Drug Pregnancy Registry found that the trend for valproate to exert a dose-dependent risk narrowly missed statistical significance.[8]

#### 6. Period of exposure and teratogenicity with valproate

Neural tube defects as a teratogenic effect can occur as early as in week 5 of gestation; that is, before most women even know that they are pregnant. Craniofacial anomalies as a teratogenic effect can occur as late as during week 22 of gestation; that is, during the second trimester, when few realize that a teratogenic risk persists.[9] Neural tube defects and craniofacial anomalies are both known to occur as teratogenic effects of valproate. Thus, there is a very wide span in pregnancy during which exposure to valproate is teratogenic.

#### 7. Valproate and IQ in the offspring

In 6- to 16-year-old children of women with epilepsy, *in utero* exposure to sodium valproate was associated with lower verbal IQ and a greater risk of mental retardation relative to *in utero* exposure to other antiepileptic drugs or no exposure to antiepileptic drugs.[10] *In utero* exposure to carbamazepine, phenytoin, or antiepileptic polytherapy was not associated with decreased IQ or a risk of mental retardation (but these conclusions were based on small subsamples). Lower maternal IQ and a larger number of tonic-clonic seizures during pregnancy were other predictors of lower verbal IQ in children born to epileptic women.[10]

### B) Prolactin and hyperprolactinemia

Answers: 1. False; 2. False; 3. True; 4. False; 5. True.

#### 1. Normal prolactin level

The normal prolactin level in a nonlactating subject is 25 ng/ml and below; adverse effects with elevated prolactin levels generally occur above levels of 30-60 ng/ml.[11] Prolactin levels are usually lower in men than in women; and in women, prolactin levels vary with the phase of the menstrual cycle.[12]

#### 2. Hyperprolactinemia and libido

Hyperprolactinemia may result in a variety of sexual adverse effects. In men, these may be decreased libido and gynecomastia. In women, these may be decreased libido, amenorrhea, infertility, breast engorgement, and galactorrhea.[11,13]

#### 3. Hyperprolactinemia and osteoporosis

Chronic hyperprolactinemia is associated with decreased levels of sex hormones and hence a decreased mineralization of bone. The resultant osteoporosis is greater in women than in men.[11,13,14]

#### 4. Hyperprolactinemia with paliperidone

Hyperprolactinemia is an important adverse effect of paliperidone. Hyperprolactinemia with paliperidone appears dose-dependent, rising to a ceiling at about 9 mg/day. Levels rise to a greater extent in women than in men.[15] Risperidone, the substituted benzamides (sulpiride, levosulpiride, and amisulpride), and typical antipsychotics also raise serum prolactin.[11,13]

#### 5. Aripiprazole augmentation for antipsychotic-induced hyperprolactinemia

Shim *et al.*[16] randomized 56 patients with haloperidol-induced hyperprolactinemia to receive either aripiprazole or placebo. During the first month, aripiprazole was dosed at 15 mg/day; and during the second month, at 30 mg/day. The haloperidol dose was held constant. At the end of the study, prolactin levels normalized in 88.5% of aripiprazole-treated patients relative to just 3.5% of placebo-treated patients. Among 11 women with menstrual disturbances in the aripiprazole group, 7 resumed menstruation during the study; none in the placebo group did. Aripiprazole treatment did not alter haloperidol levels, measures of psychosis, and measures of extrapyramidal symptoms. The important conclusion of this study is that, in patients with antipsychotic-induced hyperprolactinemia, the addition of aripiprazole can reverse the hyperprolactinemia; there is no need for the withdrawal of the offending antipsychotic drug if its continuation is deemed essential for therapeusis.
